# Metagenomic next-generation sequencing for the early diagnosis of talaromycosis in HIV-uninfected patients: five cases report

**DOI:** 10.1186/s12879-021-06551-4

**Published:** 2021-08-23

**Authors:** Qiuhua Chen, Ye Qiu, Wen Zeng, Xuan Wei, Jianquan Zhang

**Affiliations:** 1 Department of Respiratory and Critical Medicine, The Eighth Affiliated Hospital, Sun Yat-Sen University, 518000 Shenzhen, Guangdong China; 2 Department of Respiratory and Critical Medicine, The First Affiliated Hospital of Guangxi Medical University, 530021 Nanning, Guangxi China; 3 Department of Comprehensive Internal Medicine, The Affiliated Tumor Hospital of Guangxi Medical University, 530021 Nanning, Guangxi China

**Keywords:** *Talaromyces marneffei*, HIV-uninfected, Metagenomic next-generation sequencing, Diagnosis

## Abstract

**Background:**

In recent years, talaromycosis is reportedly on the rise in human immunodeficiency virus (HIV)-uninfected patients. However, the misdiagnosis and mistreatment of talaromycosis is more likely in HIV-uninfected patients than in HIV-infected patients because talaromycosis can be easily mistaken for tuberculosis or any other opportunistic infection. Therefore, we used metagenomic next-generation sequencing (mNGS), a novel gene detection method, for the diagnosis of talaromycosis in HIV-uninfected patients.

**Case presentation:**

We report five cases diagnosed as talaromycosis by mNGS in HIV-uninfected patients, which were further confirmed by tissue culture. There were 3 male and 2 female patients. Two patients had a history of rat contact. The misdiagnosis duration ranged from 88 to 245 days. While the results of tissue culture changed from repeated negative to positive, the mNGS result for *Talaromyces marneffei* was positive earlier in 4 patients. The reads of *Talaromyces marneffei* in mNGS ranged from 5 to 414. After antifungal therapy, one of the outcomes was death due to the longest duration of misdiagnosis, and the other outcomes were clinical improvement.

**Conclusions:**

mNGS is perhaps a rapid and effective diagnosis approach for the early confirmation of talaromycosis. Antifungal therapy is recommended once *Talaromyces marneffei* was revealed by mNGS. mNGS might reduce misdiagnosis duration and improve prognosis. Through these findings, we hope to provide some reference for talaromycosis in HIV-uninfected patients diagnosed early with the help of mNGS.

## Background

*Talaromyces marneffei*(TM), is a pathogenic, dimorphic fungus endemic to Southeast Asia and South China, which causes systemic mycoses involving the lungs, blood, bone marrow, skin, lymph nodes, liver, etc. [1]. An increasing number of talaromycosis have been reported in the last two decades in human immunodeficiency virus (HIV)-uninfected patients  [2]. While TM is one of the three most common opportunistic infections in HIV-infected patients, early diagnosis of talaromycosis was easy owing to specific clinical manifestations and a high rate of positive blood cultures. On the contrary, talaromycosis in HIV-uninfected patients is usually misdiagnosed as tuberculosis or another opportunistic infection [3, 4]. We found that the duration of misdiagnosis was one of the independent risk factors for death in talaromycosis patients [5]. Nevertheless, the conventional ways to diagnose talaromycosis, including fungal culture, pathological examination, or direct microscopy [3], would require a long culture period or it would be difficult to identify the thallus, which would pose challenges to early diagnosis. So far, none of rapid and effective diagnosis detections had been widely applied in clinic. Due to a high mortality rate of talaromycosis due to misdiagnosis [3, 4, 6], we considered metagenomic next-generation sequencing (mNGS), which is a novel genetic testing method for the diagnosis of microbial infections. In this study, we attempted to diagnose talaromycosis early in HIV-uninfected patients, using mNGS.

## Method

The clinical specimens were sent to the gene companies of the BGI and the Kindstar Global for different sample processing and nucleic acid extraction. DNA was extracted from body fluid specimens such as BALF after adding reagents with TIANamp Micro DNA Kit (DP316, TIANGEN BIOTECH, Beijing, China). The extracted DNA was ultrasonically broken to a fragment of about 150 bp, and the Quality Control library was constructed by Agilent 2100 Bioanalyzer and Qubit dsDNA HS Assay Kit (Thermo Fisher Scientific Inc). The platform named BGISEQ-50 / MGISEQ-2000 was used for sequencing [7]. The sequencing data of human genome were removed by BWA (BWA: http://bio-bwa.sourceforge.net/ ) [8]. After removing low complexity reads, the remaining data were compared with four microbial databases: special bacteria (6350 species), fungi (1064 species), viruses (4945 species) and parasites (234 species). The reads that can match a certain pathogen was obtained. The possible pathogens were judged according to the reads and other clinical tests.

## Case presentation

### Case 1

**Fig. 1:**
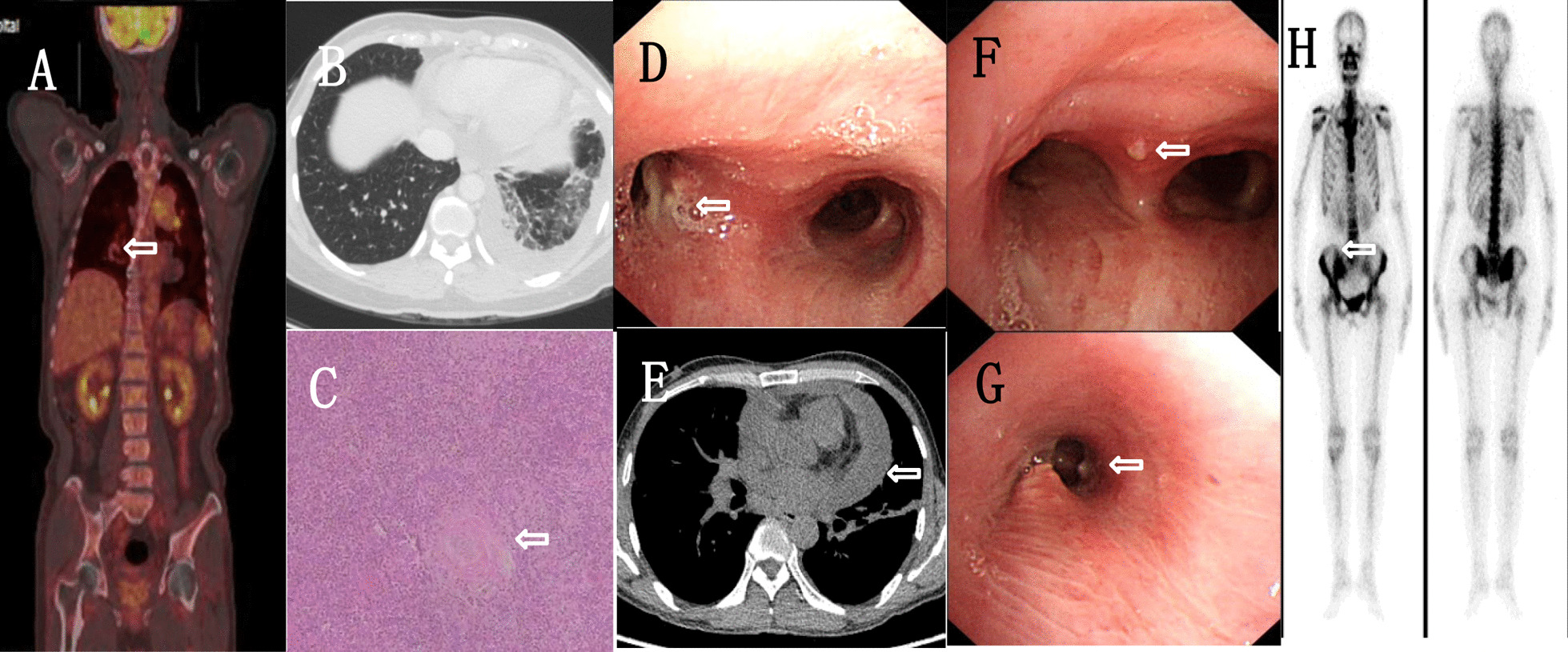
**a **Positron emission computed tomography of patient 1 revealing abnormal uptake in the lung lesions (SUVmax = 4.3), mediastinal lymph nodes (SUVmax = 14.5), cervical lymph nodes (SUVmax = 2.7), left adrenal gland (SUVmax = 3.6), cervical spine (SUVmax = 10.0) and para-aortic lymph nodes (SUVmax = 3.8). **b **Chest CT of patient 2 showing lobularinflammation.** c** Left cervical lymph node specimens from patient 2 showing chronic granulomatous inflammation of the lymph nodes with caseous necrosis. **d **Fiberoptic bronchoscopy in patient 2 showing three small nodules fused with each other at the left main bronchus. **e **Chest CT of patient 3 showing pericardial effusion. **f** Fiberoptic bronchoscopy in patient 3 showing a subcarinal tuberosity. **g** Fiberoptic bronchoscopy in patient 4 showing a nodule in the left sub-bronchus. **h** ECT of patient 4 revealing active bony metabolismin the right sacroiliac joint

A 68-year-old man, who presented with an eight-month history of prolonged fever and cough, visited our hospital on March 7, 2018. He had originally recurrent fever (Temperature max, Tmax, 39.8℃) and cough and lymphadenectasis, renal dysfunction, and emaciation followed. Lymph node specimens indicated Epstein–Barr virus associated lymphoproliferative lesion. Positron Emission Computed Tomography (PET/CT) scan showed multiple accumulations in the lungs and cervical lymph nodes (Fig. 1a). Laboratory tests revealed the following: white blood cells at 27.77 × 10^9^ cells/L; neutrophil ratio at 0.783; eosinophils at 2.99 × 10^9^ cells/L; C-reactive protein (CRP) > 192 mg/L; erythrocyte sedimentation rate (ESR) at 100 mm/h; serum creatinine at 121 µmol/L; immunoglobulin G at 33 g/L; immunoglobulin E at 393.31 IU/ml; plasma 1,3-beta glucan (G test) at 106.3 pg/ml; and HIV antibody and Galactomannan (GM) tests were negative. Tumor marker level, T lymphocyte count, CD4 + T lymphocyte count, CD8 + T lymphocyte count, and Epstein–Barr virus nucleic acid quantification level were normal. Aggravation of his condition required life support in the Intensive Care Unit (ICU), where his blood and bronchoalveolar lavage fluid (BALF) were collected for culture and mNGS, respectively, on March 15, 2018. On March 21, mNGS revealed that the reads of TM were 6 (Table 1). We administered voriconazole as antifungal therapy for 7 days (0.2 gram every 12 h (0.2 g every 12 h)). On the next day, blood culture tested positive for TM. We later replaced voriconazole with oral sequential therapy for 6 days (0.2 g every 12 h). His condition improved for some time. However, his platelet count decreased, and immune system was exhausted; T lymphocyte counts at 149; CD4 + T lymphocyte counts at 36; CD8 + T lymphocyte counts at 96; CD4:CD8 ratio at 0.38. Amphotericin B liposome was administered as antifungal therapy for 9 days (0.6 milligram/kilogram/day, mg/kg/day). He eventually died due to multiple organ failure.

### Case 2

**Fig. 2:**
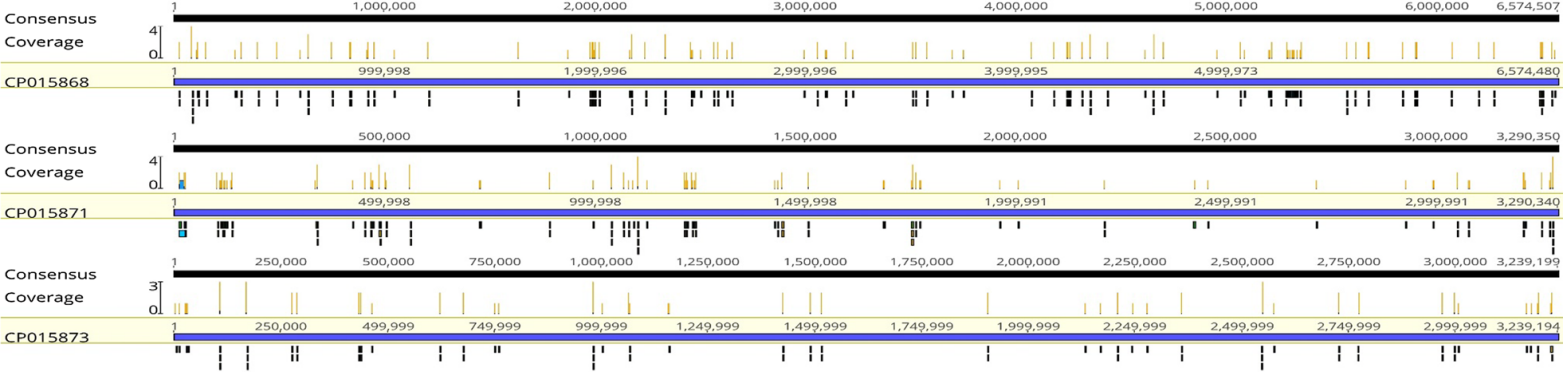
TM in mNGS in case 2 showing a high average sequence homology of 99.6%

A 43-year-old woman who suffered from fever, lymphadenopathy, and cough in the last five months, was admitted to our hospital on February 7, 2019. Initially, she had a temperature of 41℃, night sweats, swelling, and a painful cervical lymph node on the left side. Her condition improved with antibiotic therapy. Less than a month later, her left cervical lymph node was enlarged again. Pathological examination of the lymph node showed chronic granulomatous inflammation with caseous necrosis (Fig. 1c). After anti-tuberculosis treatment, her condition improved. She had coughing and wheezing on January 13, 2019, due to which she was admitted to our hospital for further treatment. Previously she had erythema nodules and took hormone therapy for a long time. Upon physical examination, moist rales were detected from the inferior lobe of the left lung. Blood testing revealed the following: white blood cells at 18.92 × 10^9^ cells/L; neutrophil ratio at 0.753; eosinophils at 0.98 × 10^9^ cells/L; ESR at 98 mm/h; CRP at 146.26 mg/L; immunoglobulin E at 227.49 IU/ml; and HIV antibody, G test, GM test results were negative. T lymphocyte count, CD4 + T lymphocyte count, CD8 + T lymphocyte count, and PCT were normal. Thoracic CT revealed left inferior lobe inflammation and left pleural effusion (Fig. 1b). Fiberoptic bronchoscope (FOB) described three small nodules were observed at the left main bronchus opening (Fig. 1d). We continued the antituberculotic treatment but she was not getting any better. On February 11, 2019, we collected BALF, subcarinal lymph node sample, blood for culture, and BALF for mNGS. On February 17, the mNGS from BALF revealed that the reads were 414 of TM (Table 1), and mean sequence homology was 99.6 % (Fig. 2). We stopped antituberculotic treatment and added voriconazole as antifungal therapy for 10 days (200 mg every 12 h, q12h). On February 20, blood culture was positive for TM. She was eventually discharged with improvement after receiving antifungal therapy for half a year under follow-up.

### Case 3

A 49-year-old man, who presented with a six-month history of prolonged fever and thoracalgia was admitted to our hospital on February 8, 2019. He had originally recurrent fever (Tmax 39.5℃), thoracalgia, and cough. His left periauricular lymph node had progressively enlarged with ulceration. Left lower lobe percussed dullness and weak breathing sounds. Blood testing revealed the following: white blood cells at 15.46 × 10^9^ cells/L; neutrophil ratio at 0.743; eosinophils at 0.79 × 10^9^ cells/L; CRP at 48.9 mg/L; and G test at 53.82 pg/ml. HIV antibody and GM test results were negative. T lymphocyte count, CD4 + T lymphocyte count, CD8 + T lymphocyte count, ESR, and PCT were normal. Thoracic CT showed pericardial effusion and left pleural effusion (Fig. 1e). FOB revealed a small nodule above the carina of the trachea (Fig. 1f). He received diagnostic anti-tuberculosis treatment because of pericardial effusion while purulent secretion cultures were negative several times. His symptoms repeated. On February 13, 2019, we collected a lymph node sample for culture and mNGS. On February 17, mNGS revealed that the reads for TM was 23 (Table 1). On February 20, blood culture was positive for TM. We administered amphotericin B liposome (0.8 mg/kg/day) for 2 weeks and voriconazole (200 mg q12h) which was replaced with itraconazole (200 mg twice a day) for economic reasons till August 1, 2019. He was still under follow-up without symptoms.

**Table 1.** Clinical manifestations and characteristics of auxiliary examinations for 5 patients

Patient	Age (years)/Sex (M/F)	Contact rodents	Misdiagnosis duration (Days)	Clinical Manifestations	Time for fungal culture (Days)	Positive specimens for fungal culture	Time for mNGS (Days)	Positive Specimens for mNGS	Reads for mNGS	Outcome
P1	68/M	No	245	Fever, cough, lymphadenectasis	7	Blood	6	BALF	6	Dead
P2	43/F	No	170	Fever, cough, lymphadenectasis	9	BALF, lymph node	6	BALF	414	Improved
P3	49/M	No	222	Fever, thoracalgia, lymphadenectasis	7	Lymph node, purulent secretion	4	Lymph node	23	Improved
P4	45/F	Yes	160	Cough, ostealgia	7	Sputum, BALF	4	BALF	5	Improved
P5	54/M	Yes	88	Fever, erythraPathological fracture	6	BALF	3	Skin	9	Improved

*M* Male, *F* Female, *BALF* Bronchoalveolar lavage fluid

### Case 4

A 45-year-old woman who suffered from right sacroiliac articulation pain and cough in last five months was admitted into our hospital on February 7, 2019. Initially, the biopsy of the right sacroiliac articulation showed chronic inflammation. Her condition improved with antibiotic treatment. Later she had cough, hemoptysis, and night sweats. She developed a large, red skin rash on her chest and back. Pain in the right sacroiliac joint percussion limited her movement. She had a history of exposure to mice. Blood testing revealed the following: white blood cells at 7.86 × 10^9^ cells/L; neutrophil ratio at 0.663; eosinophils at 0.24 × 10^9^ cells/L; and CRP at 70.86 mg/L. HIV antibody, G test, GM test results were negative. T lymphocyte count, CD4 + T lymphocyte count, CD8 + T lymphocyte count, and PCT were normal. FOB showed a nodule in the left lower lobe (Fig. 1g). Emission Computed Tomography (ECT) bone scan showed radioactive concentration in the right sacroiliac articulation (Fig. 1 h). On March 4, 2019, we collected sputum BALF for culture, and BALF for mNGS on March 5. On March 9, mNGS revealed that the reads of TM were 5 (Table 1). We administered amphotericin B liposome as antifungal therapy for 2 weeks (1.1 mg/kg/day). Blood cultures tested positive for TM on March 11, 2019. After follow-up, she continued taking voriconazole as antifungal therapy (200 mg q12h) without any symptoms of discomfort for half a year.

### Case 5

A 54-year-old man, who presented with a three-month history of prolonged fever and pustulosis, was admitted at our hospital on January 7, 2019. He had originally recurrent fever (Tmax 39℃), night sweats, and cough; he later developed pustulosis and lymphadenopathy. After 6 days, on January 4, TM was confirmed from skin culture. He was administered amphotericin B liposome as antifungal therapy. He had eaten a bamboo rat. Some moist rales were detected in the inferior lobes. Blood testing revealed the following: white blood cells at 16.21 × 10^9^ cells/L; neutrophil ratio at 0.896; PCT at 1.380 ng/ml; and G test at 244.90 pg/ml; HIV antibody and GM test results were negative. T lymphocyte, CD4 + T lymphocyte, and CD8 + T lymphocyte counts were normal. Plain film radiography showed a fracture in the left clavicle. Due to a protracted course, on January 9, we collected BALF for mNGS, which revealed the reads of TM to be 9 (Table 1), on January 12. Amphotericin B liposome treatment for antifungal therapy (0.8 mg/kg/day) and voriconazole (200 mg q12h) were continued for 25 days. Voriconazole was replaced with itraconazole (200 mg twice a day) for economic reasons till August 19, 2019 without symptoms.

## Discussion and conclusions

As we know, definite early diagnosis of talaromycosis is challenging in HIV-uninfected patients without specific clinical manifestations and a high positive rate of blood culture, thus leading to misdiagnosis and mistreatment [3, 4]. In this study, all the patients were native to the Guangxi province in south China, and two patients had a history of rat contact (Table 1). Fever, cough, thoracalgia, lymphadenopathy, ostealgia, and pathological fracture were noted (Table 1). It is difficult to identify fungal pathogens despite repeated cultures. We found that the presence of a nodule, inflammation, and tumor were observed in pulmonary imaging and PET/CT, whereas at the same time, a nodule was observed upon FOB. While lymphoproliferative lesion and caseous necrosis were observed in pathology, bone destruction was observed in ECT bone scan (Fig. 1). Cases 1–4 were diagnosed with talaromycosis by mNGS in which TM was reported as background microorganism of case 3 and 4. Later, fungal cultures further confirmed the diagnoses. Talaromycosis is often misdiagnosed as tumors, tuberculosis, or bacterial infections in the early stages of the disease. One of the outcomes was death with a long duration of misdiagnosis, and the other outcomes were clinical improvement. Also, there was another pathogen named *Staphylococcus aureus* detected by mNGS the same as blood culture (Table 2). The few reads of mNGS appeared after fungal therapy, which might explain that viable fungi decreased after therapy or opportunistic fungal infection. Therefore, it’s difficult to diagnose talaromycosis early in HIV-uninfected patients.

**Table 2.** Microorganism detected by mNGS of 5 patients

Patient	Microorganism detected by mNGS										Pathogenic microorganism except *Talaromyces marneffei*
	bacteria	reads		fungi	reads	virus		reads	others	reads	
P1	*Staphylococcus aureus*	8		*Talaromyces marneffei*	6	*Human-betaherpesvirus-5*		18	-	-	*Staphylococcus aureus*
P2	–	–		*Talaromyces marneffei*	414	*Human-betaherpesvirus-5*		6	–	–	–
						*Human-gamma herpesvirus-4*		2			
P3	–	–		–	–	–		–	–	–	–
P4	–	–		*Pneumocystis–jirovecii*	7	–		–	–	–	–
P5	*Veillonella parvula*		190	*Talaromyces marneffei*	9	*Human gammaherpesvirus 4*	2		–	–	–
	*Veillonella atypica*		149								
	*Streptococcusparasanguinis*		94								
	*Streptococcus australis*		13								
	*Rothia aeria*		55								
	*Rothia mucilaginosa*		46			*Torque teno virus*	2				
	*Prevotella salivae*		39								
	*Prevotellamelaninogenica*		28								
	*Lautropia mirabilis*		87								
	*Lactobacillus salivarius*		37								

We regarded fungal culture as the gold standard for the diagnosis of talaromycosis, which required a long incubation period and was often not obtained. Recently, we found other techniques, such as polymerase chain reaction (PCR), nested PCR, enzyme-linked immunosorbent assay (ELISA), rapid lateral flow immunochromatographic test (ICT), and mNGS, that allow early diagnosis [9–11]. mNGS, one of the high-throughput sequencing methods, is a new technology that allows millions of DNA molecules to be sequenced simultaneously. It integrates all the nucleic acid sequences and isolates the microorganism and host sequences from them, to analyze the reads of microorganisms to identify the species and the abundance of microorganisms. Finally, the causative pathogen is confirmed [12, 13]. This method has been successfully used to identify unexplained infections, including blood, respiratory, and gastrointestinal infections [13, 14]. Fungal infections have rarely been diagnosed using mNGS [13]. Compared to other molecular techniques, mNGS is expensive but quick and convenient and operability. PCR-based molecular diagnostic techniques are based on the gene sequences of known pathogens for targeted detection, and the positive rate of clinical diagnosis is low for complex pathogens. However, mNGS, as a new high-multiplex PCR technique in a broad sense, can extract all microbial nucleic acid sequences for rapid diagnosis. Therefore, it’s more valuable as opposed to direct PCR. As we know, further improvement is needed in formulating standardized procedures, unifying diagnostic standards and databases of major laboratories, and gradually reducing high testing costs, but it is likely to become a necessary means of clinical microbiological diagnosis in the future [15]. At the moment mNGS can’t distinguish between real pathogens and colonizing bacteria fully. But the nucleic acid sequence alignment is based on the four basic microbial genome databases of bacteria, fungi, virus and parasites in national center of biotechnology information (NCBI), including a variety of special pathogens. The summary and update of colonization bacteria in different human organs, sampling environment and laboratory background microorganisms will help us distinguish normal flora and pollution in the detection process of mNGS before identifying pathogenic microorganisms. Besides TM isn’t colonization of fungi in lung. Davidiellaceae and Cladosporium were reported as the main fungi flora in healthy BALF [16]. Therefore, the diagnostic value for talaromycosis in HIV-uninfected patients’ early stage could be a great help once TM is found via mNGS, even it’s regarded as a background microorganism in the reports of mNGS.

Previously a case had been successfully diagnosed using mNGS [11]. Here we tried to diagnose talaromycosis early in HIV-uninfected patients with the help of mNGS. In the five cases, the reads of mNGS ranged from 5 to 414. In our opinion, high reads are indicative of a high TM content in the tissue samples, and low reads indicate a low content of viable fungi, which may be easily regarded as background microorganisms and is filtered. Certainly, the database and data processing methods of different sequencing platforms need to be considered. mNGS still can’t be a routine testing for economic reasons in developing countries but we suggest that the early use of mNGS is recommended while conducting tissue culture when HIV-uninfected patients present with recurrent fever, cough, lymphadenopathy, repeated negative cultures, and no response to antibiotics or diagnostic anti-tuberculosis, especially with abnormal immune function [17]. We suggest BALF collection, lymph node, skin, or bone sample collection for mNGS. With respect to the results of mNGS, we believe the reads were related to the sampling position. The detection of TM can’t be ignored with low reads. At this point experimental antifungal decision should be made prior to culture results once TM was found in mNGS result. Early targeted antifungal therapy can improve prognosis of talaromycosis in HIV-uninfected patients.

Overall, mNGS is perhaps the relatively early, rapid, and effective diagnosis method, which might improve the rate of confirmed diagnosis of talaromycosis in HIV-uninfected patients. We suggest that the early use of mNGS is recommended when HIV-uninfected patients present with recurrent fever and no response to conventional treatments. The diagnostic value for talaromycosis in HIV-uninfected patients’ early stage could be a great help once *Talaromyces marneffei* is identified via mNGS. Early targeted antifungal therapy can improve prognosis. We hope that this study provides some reference for talaromycosis in HIV-uninfected patients diagnosed early by mNGS.

## Data Availability

All data generated or analysed during this study are included in this published article.

## Abbreviations

BALF: Blood and bronchoalveolar lavage fluid
CRP: C-reactive protein
ECT: Emission Computed Tomography
ESR: Erythrocyte sedimentation rate
g: Gram
G test: Plasma 1,3-beta glucan
GM test: Galactomannan test
HIV: Human immunodeficiency virus
h: Hour
ICU: Intensive Care Unit
kg: Kilogram
mNGS: Metagenomic next-generation sequencing
mg: Milligram
PET/CT: Positron Emission Computed Tomograph
Tmax: Temprature max
